# The Impact of Inflammatory Markers and Obesity in Chronic Venous Disease

**DOI:** 10.3390/biomedicines12112524

**Published:** 2024-11-04

**Authors:** Flavia-Medana Petrascu, Sergiu-Ciprian Matei, Mădălin-Marius Margan, Ana-Maria Ungureanu, Gheorghe-Emilian Olteanu, Marius-Sorin Murariu, Sorin Olariu, Catalin Marian

**Affiliations:** 1 Department of Doctoral Studies, “Victor Babeș” University of Medicine and Pharmacy, 300041 Timișoara, Romania; flavia.petrascu@umft.ro (F.-M.P.); olteanu.gheorghe@umft.ro (G.-E.O.); cmarian@umft.ro (C.M.); 2 Department of Biochemistry, “Victor Babeș” University of Medicine and Pharmacy, 300041 Timisoara, Romania; 3 Abdominal Surgery and Phlebology Research Center, “Victor Babeș” University of Medicine and Pharmacy, 300041 Timișoara, Romania; murariu.marius@umft.ro (M.-S.M.); olariu.sorin@umft.ro (S.O.); 4 1’st Surgical Department, Pius Brînzeu Emergency County Hospital, 300723 Timișoara, Romania; 5 Department of Functional Sciences, Discipline of Public Health, “Victor Babeș” University of Medicine and Pharmacy, 300041 Timisoara, Romania; margan.madalin@umft.ro; 6 Center for Translational Research and Systems Medicine, Faculty of Medicine, “Victor Babeș” University of Medicine and Pharmacy, 300041 Timișoara, Romania; 7 Department of Radiology and Medical Imaging, “Victor Babeș” University of Medicine and Pharmacy, 300041 Timisoara, Romania; ungureanu.ana-maria@umft.ro; 8 Research Center for Pharmaco-Toxicological Evaluations, Faculty of Pharmacy, “Victor Babeș” University of Medicine and Pharmacy, 300041 Timisoara, Romania; 9 Center for Research and Innovation in Personalized Medicine of Respiratory Diseases, “Victor Babeș” University of Medicine and Pharmacy, 300041 Timisoara, Romania; 10 Center for Complex Networks Science, “Victor Babeș” University of Medicine and Pharmacy, 300041 Timisoara, Romania

**Keywords:** obesity, inflammation, chronic venous disease, endothelial dysfunction, microcirculatory damage

## Abstract

**Background:** Chronic venous disease (CVD) represents a significant health challenge, particularly in obese individuals. This study focuses on the interplay between inflammation, obesity, and CVD, by analyzing the role of inflammatory markers in the disease progression. **Methods:** Clinical and paraclinical data of 619 patients hospitalized and treated in the Phlebology Department (1stSurgical Department, “Pius Brînzeu” Emergency County Hospital Timișoara, Romania) between 2018 and 2024 were analyzed. **Results:** The statistical analysis revealed that age, C-reactive protein (CRP), fibrinogen, and absolute neutrophil count (ANC) were key predictors of CVD progression. Specifically, elevated CRP and fibrinogen levels correlated strongly with increased CVD severity, particularly in patients with higher body-mass index (BMI). BMI, while not an independent predictor, contributed indirectly to the disease severity through its association with these inflammatory markers. The logistic regression model incorporating age, BMI, CRP, fibrinogen, and ANC demonstrated a high predictive accuracy, with an area under the curve (AUC) of 0.902, highlighting the models reliability in stratifying patients at risk for severe CVD. **Conclusions:** This predictive model not only aids in identifying high-risk patients but also reinforces inflammation as a critical therapeutic target in CVD management.

## 1. Introduction

One of the major problems affecting the overall health of the population worldwide is the pathological excess of adipose tissue, leading to a high body mass index (BMI), commonly referred to as obesity [1]. As of 2022, according to the World Health Organization, 890 million adults are dealing with obesity, representing approximately 16% of the global adult population. This represents a substantial increase in the number of obese individuals since 1990, making obesity a significant health concern [2]. As observed in other countries, obesity has increased in Romania as well. Scattered meals and a sedentary lifestyle are some of the factors contributing to the rise of obesity in most countries [3].

Metabolic and cardiovascular diseases are established to be associated with structural changes generated by obesity. However, another equally important part associated with obesity is the dysregulation of the immune system [4,5]. In obese patients, inflammation extends beyond the adipose tissue, a consequence of the constant release of pro-inflammatory molecules by the adipose cells [6]. Additionally, as adipocytes enlarge, they may undergo cellular stress due to hypoxia, which triggers the release of danger signals and pro-inflammatory mediators, contributing to the overall inflammatory response [7]. In obese individuals, stimuli such as free fatty acids and reactive oxygen species (ROS) lead to NF-κβ-mediated inflammatory cascade activation and subsequent release of the pro-inflammatory cytokines [8,9]. The NF-κβ pathway has a key role in inflammation by mediating IL-6, tumor necrosis factor-alpha (TNF-α), and other cytokines, along with the polarization of the macrophages to type M1. While M2 macrophages facilitate tissue remodeling and angiogenesis, M1 macrophages drive pro-inflammatory responses [10].

Chronic inflammation in obesity promotes fibrosis within the adipose tissue, altering its architecture and exacerbating the inflammatory state. The process of angiogenesis, essential for accommodating the expanding adipose tissue, may become dysfunctional. Inadequate and dysfunctional blood vessels contribute to hypoxia and immune cell infiltration, further fueling the inflammation [11].

Along with the inflammation, obesity significantly exacerbates venous stasis through various mechanical factors. The low physical activity and, consequently, the diminished effectiveness of the muscle pump can contribute to venous stasis. Additionally, excess body weight can reduce the efficiency of venous valves and compress the leg veins, increasing the risk of varicose veins and worsening the venous stasis [12].

In addition, the inflammatory signals pervade throughout the entire body via the bloodstream, described in obesity as “chronic low-grade inflammation”. Chronic inflammation in obesity alters the function of the endothelium, increasing the leukocyte migration and vascular wall permeability. The outcome of the impaired endothelium function is an important etiologic factor that contributes to a multitude of diseases; an important one is chronic venous disease (CVD) [13].

CVD is a prevalent global issue, impacting the quality of life for 60–70% of the population [14]. Given this substantial occurrence locally and globally, CVD is undeniably a common pathology, with disease severity and treatment requirements escalating with age. A retrospective study in the United States indicates that initial symptoms like lower limb pain, heaviness, and general fatigue are more common in patients under 65, while more severe manifestations and more elaborate treatments are prevalent in patients aged 65 and older [15]. In Romania, recent findings reveal a high prevalence of 68.4%, marking a significant increase from a study conducted a decade ago [16,17].

The structural and functional changes that damage the venous endothelium are predominantly occurring in the lower extremities. The symptoms may vary from mild (telangiectasia, varicose veins) to more severe (edema, skin changes, and ulcers). The ongoing treatment for CVD includes conservative treatment, as well as surgical management. The main element in conservative treatment is represented by compression stockings, which serve more as preventive care. In the case of obesity, the management of weight loss is prioritized. The surgical arsenal includes diverse methods; depending on the location and etiology of the problem, insufficient veins are either occluded or removed during these procedures. Modern options like classic stripping or cryostripping and endovenous thermal ablation are safe, with low postoperative morbidity and excellent cosmetic results [18,19,20].

The inflammation generated by obesity may play a substantial part in the dysregulation of the endothelium in CVD. The aim of the study is to identify the correlation between inflammatory markers and the severity of CVD in obese patients. To reach the objectives of the study, we use serological markers (complete blood count, inflammatory markers, coagulation factors, and biochemistry routine parameters) to construct the optimal inflammatory parameter cluster that can predict CVD evolution.

## 2. Materials and Methods

Patients and laboratory tests. This prospective study monitored the variations of serological laboratory parameters correlated with BMI in CVD patients. The blood test results and clinical data of 672 patients who were admitted for CVD treatment in the Phlebology Department (1st Surgical Department, “Pius Brînzeu” Emergency County Hospital Timișoara, Romania) between 1 October2018 and 31 May2024 were evaluated (all patients who presented with CVD and signed the inform consent of participating in the study during the defined time period were initially included). The diagnosis of CVD was established in a clinical setting and was completed by ultrasound examination. All the patients were evaluated before surgery by a ‘pulse-wave’ Doppler exam, with venous reflux being noted (GE Healthcare Venue40 Doppler ultrasound with a 12L-SC transducer was used, Wauwatosa, WI, USA). Venous Doppler ultrasound is the method of choice in venous vascular pathology as it is quickly performed, easily accessible, noninvasive, and non-ionizing. For venous disease assessment, duplex ultrasound with segmental compression every 2 cm was used, with the patient in a supine position with a slightly elevated head. We used “frog-leg position” (externally rotated legs, bent knees) and ultrasound examination from the common femoral vein at the inguinal ligament to the distal veins, using a linear probe. All the patients were evaluated using the same ultrasound unit and the same environmental conditions. The degree of flow obstruction was evaluated by color Doppler examination and thrombus characterization using grayscale protocol.

For the laboratory tests, venous blood samples were collected from the elbow or anterior antebrachial region veins. All of the analyzed data refer to the first ambulatory presentation of each patient. The following data were collected: age; body mass index (BMI); basic laboratory tests, including complete blood count (CBC) and inflammatory markers including red blood cell count (RBC) and white blood cell count (WBC) with WBC differential (neutrophils, lymphocytes, eosinophils, basophils, and monocytes), platelet count (PLT); erythrocyte sedimentation rate (ESR); C-reactive protein (CRP); fibrinogen; coagulation factors, including prothrombin time (in percentages and seconds), international normalized ratio (INR), and activated partial thromboplastin time (aPTT); characteristic markers for muscle tissue, including creatine kinase (CK) and CK myocardial band (CK-MB); and hepatic and metabolic markers, including alanine transaminase (ALT), aspartate transaminase (AST), and glycemia. The laboratory reference values for the studied parameters were as follows: RBC, 4.5–5.9 × 10⁶/µL; WBC, 4–9.5 × 10^3^/µL; neutrophils percentage, 45–70%; lymphocytes percentage, 20–40%; eosinophils percentage, 0–4%; basophils percentage, 0–1%; monocytes percentage, 3.5–9.5%; PLT, 150–400 × 10^3^/µL; ESR, 0–15 mm/h; CRP, 0–10 mg/L; fibrinogen, 200–393 mg/dL; prothrombin time (PT) percentage, 90–148%; PT, 9.4–12.5 s; INR, 0.85–1.14; aPTT, 25.1–36.5 1/s; CK, 30–170 U/L; CK-MB, 0–16 U/L; glycemia, 74–106 mg/dL; and ALT, 0–55 U/L; AST, 5–34 U/L.

Enrollment criteria and study groups. In order to establish uniformity in the groups, the following data were excluded from the study: patients who did not sign the written informed consent form in order to participate in the study; patients with confirmed acute or chronic inflammatory diseases (including acute thrombophlebitis, deep vein thrombosis, asthma, inflammatory bowel disease), and patients who followed a treatment with anti-inflammatory medicines recently within the previous 4 weeks. After applying these criteria, 619 patients were included in the study. These patients were assigned to three groups according to the stage of the disease, using the clinical aspect (C) from the Clinical, Etiological, Anatomical, and Pathophysiological (CEAP) classification [21]: Group 1 (milddisease) included 251 patients in stages C2 and C3, Group 2 (moderate to severedisease) included 214 patients in the C4 stage, and Group 3 (severedisease) included 154 patients in stages C5 and C6. Subsequently, each group of patients was divided according to the body mass index in normal weight patients (BMI: 18.5 to 24.9 kg/m^2^), overweight patients (BMI: 25.0 to 29.9 kg/m^2^), and obese patients (BMI: >30.0 kg/m^2^). The obese patients were evaluated according to the World Health Organization criteria, which further classifies obesity into three classes: Class I (BMI: 30.0 to 34.9 kg/m^2^), Class II (BMI: 35.0 to 39.9 kg/m^2^), and Class III (BMI: ≥40.0 kg/m^2^) [22].

Statistical analysis. All patient data was collected and processed in the MediFlux Trusted Research Environment (Originis^TM^, Amsterdam, The Netherlands). Statistical analysis was conducted using R version 4.2.0 (R Foundation for Statistical Computing, Vienna, Austria). Data manipulation was performed using the R “base” package, while figures and graphs were plotted using the “ggplot” package. Statistical tests, univariate analyses such as linear and logistic regression, as well as multivariate analyses including multiple logistic regression, were executed using the R “stats” package. The “pROC” package facilitated the analysis and visualization of Receiver Operating Characteristic (ROC) curves, including computing their confidence intervals and conducting hypothesis tests.

Continuous variables were described using means and standard deviations, and categorical variables were reported as counts and percentages. The non-parametric Kruskal–Wallis H test was employed to compare multiple independent samples that did not adhere to a normal distribution for numerical variables, while the Chi-square test was applied to categorical variables. ROC analysis was used to evaluate the capacity of clinical and paraclinical features to differentiate between disease severity groups, identifying the appropriate cut-off values.

The association between the dependent and predictor variables was examined using statistical tests with a significance threshold set at 0.05. No imputation of missing data was conducted during the statistical analysis.

## 3. Results

This study analyzed the connection between obesity and chronic venous disease (CVD) through a range of clinical and paraclinical parameters. Out of the 672 patients initially screened, 619 met the inclusion criteria, and the data for these patients were collected and analyzed. Patients were divided based on CVD severity into three groups: mild (N = 251), moderate (N = 214), and severe (N = 154). The distribution of patients based on BMI was also included for the correlation with inflammatory markers.

### 3.1. Clinical and Paraclinical Characteristics

Table 1 shows the division of the patients into three groups based on CVD severity: mild (N = 251), moderate (N = 214), and severe (N = 154). The average age increases with the severity of CVD, with severe cases having a mean age of 65.73 years compared to 54.51 years in mild cases. This difference is statistically significant (*p* < 0.001), indicating that older age is associated with more severe CVD. The proportion of females is higher in all severity groups. In the mild group, 74.10% are female compared to 25.90% male. In the severe group, 70.78% are female and 29.22% male. This difference is statistically significant (*p* = 0.003). BMI also increases with CVD severity, with the moderate group having a mean BMI of 30.04 compared to 26.75 in the mild group. This difference is statistically significant (*p* < 0.001), suggesting a strong association between higher BMI and more advancedCVD progression. Higher BMIs are more prevalent in severe CVD cases. For example, 22.07% of patients in the severe group have Class I Obesity, 16.88% have Class II Obesity, and 2.59% have Class III Obesity. In contrast, only 15.93% of the patients from the mild group have Class I Obesity, and 0.39% have Class II Obesity. These differences are statistically significant (*p* < 0.001). The red blood cell (RBC) count is lower in severe cases, while the white blood cell (WBC) count is higher. Both statistically significant differences (*p* < 0.001) indicate an inflammatory response. Absolute neutrophil count (ANC) increases significantly with CVD severity, with severe cases having a mean ANC of 5.240 compared to 3.979 in mild cases (*p* < 0.001). Absolute lymphocyte count (ALC) decreases as CVD severity increases, with severe cases having a mean ALC of 1.750 compared to 2.084 in mild cases (*p* < 0.001). C-reactive protein (CRP) and fibrinogen levels rise significantly with the severity of CVD. The severe group has a mean CRP of 21.083 compared to 5.098 in the mild group and a mean fibrinogen level of 419.500 compared to 318.980 in the mild group (both *p* < 0.001). Elevated erythrocyte sedimentation rate (ESR) and fasting glucose levels also correlate with increased CVD severity (both *p* < 0.001).

As seen in Table 2, age is strongly correlated with severe CVD, with a Spearman correlation coefficient of 0.411 and a *p*-value of <0.001. Each additional year of age increases the odds of severe CVD by approximately 11.6%, as indicated by the crude odds ratio (cOR) of 1.116 (95% CI: 1.092–1.142). BMI is also positively correlated with severe CVD, with a Spearman correlation coefficient of 0.109 and a *p*-value of 0.006. The cOR for BMI is 1.062 (95% CI: 1.023–1.104), indicating that higher BMI increases the odds of severe CVD, though less strongly than age. The female sex (as an individual risk factor) does not show a significant correlation with severe CVD, with a Spearman correlation coefficient of 0.030 and a *p*-value of 0.453.

The correlation between paraclinical predictive factors and severe form highlights the strong positive correlation between severe CVD and inflammatory markers (ANC, CRP, and fibrinogen) (see Table 3). The Spearman correlation coefficients for ANC, CRP, and fibrinogen are 0.189, 0.456, and 0.324, respectively, with *p*-values of <0.001 for all three. The crude odds ratios (cOR) are 1.288 (95% CI: 1.169–1.424) for ANC, 1.140 (95% CI: 1.112–1.171) for CRP, and 1.007 (95% CI: 1.005–1.009) for fibrinogen, indicating that higher levels of these markers are significantly associated with severe CVD. White blood cell (WBC) count and erythrocyte sedimentation rate (ESR) also correlate positively with severe CVD. The Spearman correlation coefficient for WBC is 0.078 (*p* < 0.001) and for ESR is 0.473 (*p* < 0.001), with cORs of 1.113 (95% CI: 1.026–1.207) and 1.089 (95% CI: 1.070–1.110), respectively. Other markers like RBC, ALC, and CK show varying degrees of correlation, with RBC and ALC being negatively correlated with severe CVD.

In the multivariate analysis (see Table 4), we take into consideration predictors—age, BMI, CRP, fibrinogen, and ANC—over other parameters based on their consistent and significant association with CVD severity, which we previously mentioned. Age, CRP, fibrinogen, and ANC remain significant independent predictors of severe CVD. The adjusted odds ratio (aOR) for age is 1.101 (95% CI: 1.072–1.130) with a *p*-value of <0.001, indicating a strong independent effect of age on CVD severity. CRP has an aOR of 1.218 (95% CI: 1.164–1.275) with a *p*-value of <0.001, making it a robust predictor of severe CVD. Fibrinogen aOR is 1.003 (95% CI: 1.0002–1.0058) with a *p*-value of 0.033, indicating that higher fibrinogen levels are independently associated with increased severity of CVD, although the effect size is smaller compared to CRP. ANC has an aOR of 1.333 (95% CI: 1.170–1.520) with a *p*-value of <0.001, emphasizing its significant role as an inflammatory marker linked to severe CVD.

Although BMI was included in the multivariate analysis, its aOR was 0.764 (95% CI: 0.702–0.830) with a *p*-value of <0.001, revealing a counterintuitive negative association with severe CVD. The VIF (Variance Inflation Factor) results show higher values for BMI (2.500) and CRP (2.276), indicating a moderate level of collinearity between these two variables, likely due to their roles in inflammation and metabolic factors. However, since the VIF values are still below the common threshold of 5, there is no indication of severe multicollinearity.

To further clarify this finding, a multivariate analysis of the combined moderate and severe CVD forms was conducted to capture a broader range of disease severity, allowing for a more comprehensive understanding of how predictive factors influence CVD progression (see Table 5).

While age, CRP, fibrinogen, and ANC remain statistically significant predictors of CVD severity in the combined analysis of moderate and severe forms, BMI did not retain statistical significance (aOR = 1.024, 95% CI: 0.973–1.078, *p* = 0.357). This suggests that when adjusting for other key inflammatory and demographic factors, BMI alone does not independently predict the progression of more severe CVD. The lack of statistical significance for BMI in this context could indicate that its influence on CVD severity may be mediated through other factors, such as inflammation (represented by CRP and fibrinogen), rather than acting as a direct, independent predictor. Furthermore, for this model, there is no strong collinearity between BMI and CRP (VIF values of 1.339 and 1.311).

Nevertheless, when categorizing CVD patients into two main groups—normal weight and overweight (including obese patients)—a clearer distribution of these categories emerges across the different severity levels of CVD (see Table 6). As the severity of CVD progresses, we observe an increasing proportion of overweight and obese patients while the percentage of normal-weight patients declines. Specifically, among patients with mild CVD, 72.11% are overweight or obese, and 27.89% are of normal weight. This proportion becomes more pronounced in the moderate CVD group, where 77.1% are overweight or obese, and only 22.89% are normal weight. Finally, in the severe CVD group, 82.46% are overweight or obese, with just 17.53% falling into the normal weight category.

While BMI was not considered a statistically significant risk factor in the multivariate regression analysis for predicting CVD severity (aOR = 1.024, 95% CI: 0.973–1.078, *p* = 0.357), the high prevalence of overweight and obese patients in the more severe CVD groups cannot be overlooked. As shown in Table 6, the percentage of overweight and obese patients consistently increases as the severity of CVD progresses. This trend suggests that BMI might still play an indirect role in CVD progression despite not achieving statistical significance in the multivariate correlation. This led us to include BMI as a predictive factor, acknowledging its high representation among patients with advanced disease.

### 3.2. Inflammatory Markers and Disease Severity

**Figure 1:**
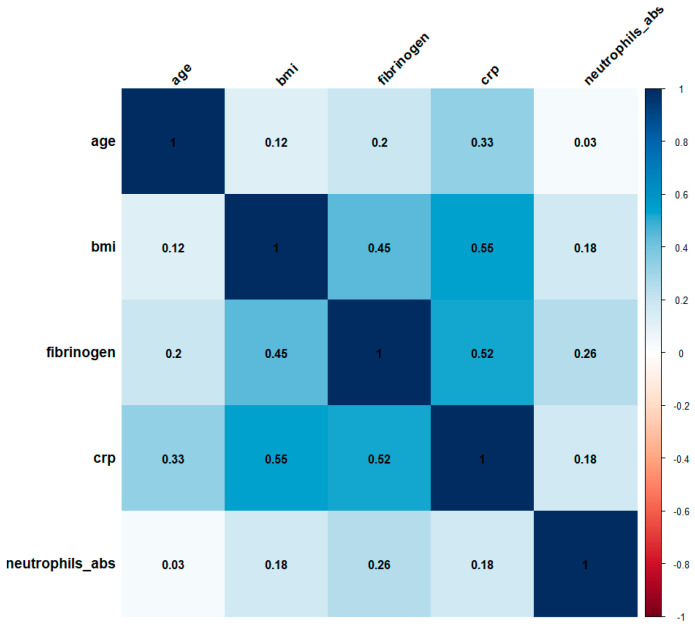
Correlation Matrix between predictive factors of CVD.

Figure 1 illustrates the correlation matrix between key predictive factors, such as age, BMI, CRP, fibrinogen, and ANC. The correlation matrix shows the strength of the relationship between pairs of variables. BMI strongly correlates with CRP, with a correlation coefficient (r) of 0.55 and a fibrinogen correlation coefficient (r) of 0.45.

Age shows a moderate positive correlation with CRP (r = 0.33), indicating that older patients tend to have higher CRP levels, possibly contributing to increased CVD severity. The correlation between age and fibrinogen is weaker (r = 0.20), and the correlation with ANC is very weak (r = 0.03). CRP (C-reactive protein) stands out with strong positive correlations with BMI (r = 0.55), fibrinogen (r = 0.52), and age (r = 0.33). This highlights CRP as a significant inflammatory marker influenced by both BMI and age.

Fibrinogen has moderate positive correlations with BMI (r = 0.45) and CRP (r = 0.52) and a weaker correlation with ANC (r = 0.26). These correlations suggest that fibrinogen, another important inflammatory marker, is also influenced by BMI and CRP levels. ANC (absolute neutrophil count) shows a moderate correlation with fibrinogen (r = 0.26) and weak correlations with BMI (r = 0.18) and CRP (r = 0.18). This indicates that ANC is less influenced by BMI compared to CRP and fibrinogen but still plays an important role in the inflammatory response associated with CVD.

**Figure 2:**
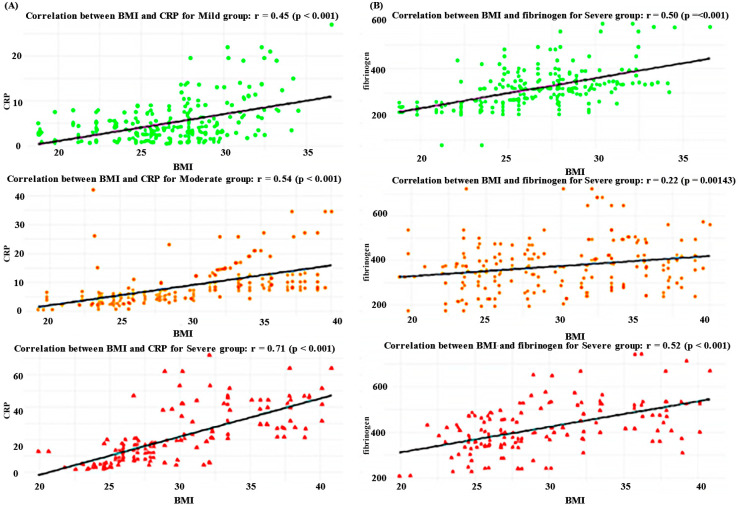
Correlation between BMI and Inflammatory Markers (CRP and fibrinogen) stratified by disease severity: mild, moderate and severe groups. Abbreviations: r—correlation coefficient; *p*-value ≤ 0.001. (**A**) Correlation between BMI and CRP illustrated across three disease severity groups: mild (r = 0.45, *p* < 0.001), moderate (r = 0.54, *p* < 0.001), and severe (r = 0.71, *p* < 0.001). (**B**) Correlation between BMI and fibrinogen, with the mild group showing a moderate positive correlation (r = 0.55, *p* < 0.001), the moderate group showing a weaker positive correlation (r = 0.22, *p* = 0.00143), and the severe group showing a moderate positive correlation (r = 0.52, *p* < 0.001).

Further, we wanted to display the relationship between CRP and BMI, and fibrinogen and BMI, to evaluate whether BMI correlates with these inflammatory markers. Figure 2 displays multiple linear regressions between BMI and key inflammatory markers, considering the severity of chronic venous disease. Panel A shows the relationship between BMI and CRP, while Panel B illustrates the correlation between BMI and fibrinogen levels in all severity groups.

The scatter plots and linear regression lines in Figure 2 demonstrate that BMI exhibits a strong positive correlation with CRP in the mild (r = 0.45, *p* < 0.001), moderate (r = 0.54, *p* < 0.001), and severe (r = 0.71, *p* < 0.001) groups. This strong correlation is also displayed in correlation with fibrinogen in the mild (r = 0.50, *p* < 0.001), moderate (r = 0.22, *p* < 0.001), and severe (r = 0.52, *p* < 0.001) groups.

**Figure 3:**
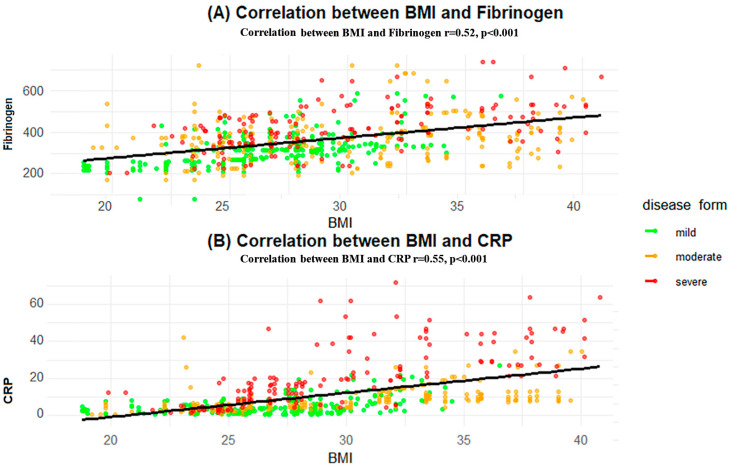
Correlation between BMI and inflammatory biomarkers stratified by CVD Severity. (**A**) The X-axis represents BMI (kg/m^2^), and the Y-axis represents fibrinogen levels (mg/dL); Correlation between BMI and fibrinogen levels, with a correlation coefficient of r = 0.52 and *p* < 0.001. (**B**) The X-axis represents BMI (kg/m^2^), and the Y-axis shows C-reactive protein (CRP) levels (mg/L);positive correlation between BMI and CRP levels, with a correlation coefficient of r = 0.55 and *p* < 0.001.

In Figure 3A shows the relationship between BMI and CRP, with r = 0.55 and *p* < 0.001, indicating a strong positive correlation. As observed in the scatter plot, the upward slope of the trend line reinforces the correlation between the CRP level and that this relationship strengthens with the increase of the CVD severity.

In Figure 3B, the relationship between BMI and fibrinogen is shown, with r = 0.52 and *p* < 0.001, also demonstrating a moderate positive correlation. While the association between BMI and fibrinogen is not as strong as that between BMI and CRP, the upward trend indicates that higher BMI is associated with increased fibrinogen levels.

### 3.3. Predictive Models for Severe CVD

**Figure 4:**
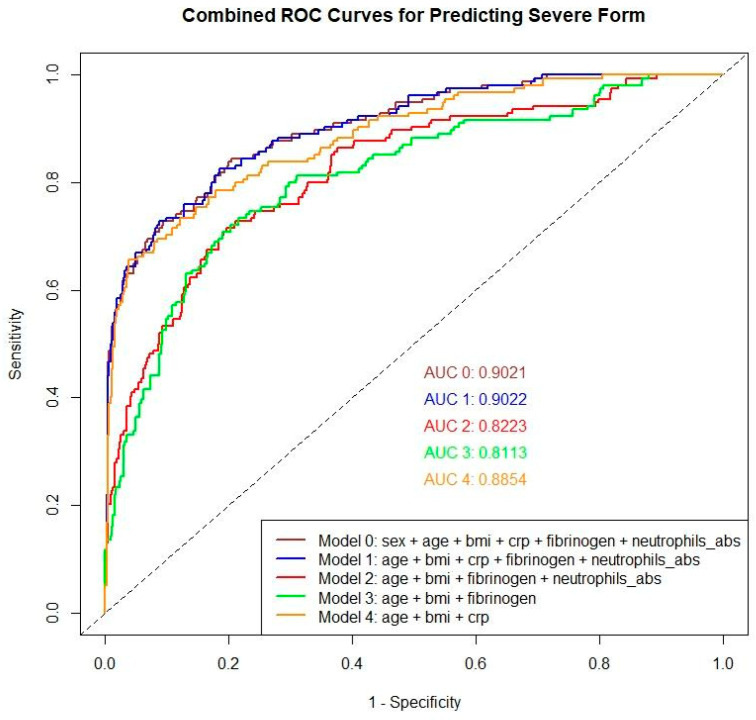
ROC Curves models predicting Severe Form.

With the scope of detecting predicting levels contributing to the development of severe form, five new logistic regression models were developed, represented in Figure 4, incorporating two clinical parameters (age and BMI) and three paraclinical parameters (inflammatory markers—CRP, ANC, and fibrinogen) as predictors. Model 1 included five factors: age, body mass index (BMI), C-reactive protein (CRP), fibrinogen, and absolute neutrophil count (ANC) with the logistic regression equation: logit(p) = −3.9538 + 0.0958119 × age − 0.269699 × BMI + 0.197272 × CRP + 0.00304332 × fibrinogen + 0.0028749 × ANC. The ROC curve analysis for this model shows an AUC of 0.902 (95%CI: 0.874), with a cut-off value of 0.219, sensitivity of 82.5%, and specificity of 81.5%. Model 2 uses age, BMI, fibrinogen, and ANC as variables, described by the equation: logit(p) = −10.4111 + 0.108991 × age − 0.0187926 × BMI + 0.00560958 × fibrinogen + 0.00238548 × ANC, resulting in an AUC of 0.822 (95%CI: 0.783), a cut-off value of 0.259, sensitivity of 72.7%, and specificity of 78.9%. Model 3, formulated with age, BMI, and fibrinogen, is expressed as logit(p) = −9.46182 + 0.105275 × age − 0.0137796 × BMI + 0.00631186 × fibrinogen, showing an AUC of 0.811 (95%CI: 0.770), with a cut-off value of 0.261, sensitivity of 73.4%, and specificity of 78.4%. Lastly, Model 4, which includes age, BMI, and CRP, follows the equation: logit(p) = −2.13214 + 0.0863019 × age − 0.225287 × BMI + 0.199233 × CRP, with a ROC curve yielding an AUC of 0.885 (95%CI: 0.854), a cut-off value of 0.235, sensitivity of 78.6%, and specificity of 82.1%.

Four logistic regression models were developed to predict the development of severe CVD using a combination of clinical and paraclinical parameters. Model 1, which included age, BMI, CRP, fibrinogen, and ANC, with an AUC of 0.9022, with sensitivity of 82.5% and specificity of 81.5%. Model 2 incorporated age, BMI, fibrinogen, and ANC, resulting in an AUC of 0.8223, sensitivity of 72.7% and specificity of 78.9%. Model 3 used age, BMI, and fibrinogen, with an AUC of 0.8113, sensitivity of 73.4% and specificity of 78.4%. Model 4 included age, BMI, and CRP, with an AUC of 0.8854, sensitivity of 78.6% and specificity of 82.1%.

Among these models, Model 1 performs the best due to its high AUC, sensitivity and specificity, indicating the most accurate prediction of severe CVD based on the provided data. Interestingly, Model 0, which includes the patient’s sex in addition to the other factors from Model 1, results in an AUC of 0.9021, nearly identical to Model 1, but it does not outperform it. Model 4 also performs well but slightly lower than Model 1, suggesting that the inclusion of CRP without fibrinogen does not enhance the predictive power as much as the combination of them in Model 1.

## 4. Discussion

Based on the statistical analysis, the following factors: age, CRP, fibrinogen, and ANC were found to be the most significant predictors independently linked to the progression of CVD severity. While BMI correlates positively with CVD severity, it is not an independent risk factor when age, CRP, fibrinogen, and ANC are accounted for. Both CRP and fibrinogen showed an increased correlation with BMI as CVD severity progresses, particularly CRP. The strong correlation between BMI and CRP, especially in severe CVD, suggests potential confounding in multivariate analyses where both variables are included. This explains why BMI may not emerge as an independent predictor when inflammatory markers like CRP are considered. This could indicate that the impact of BMI on CVD severity is largely mediated through its effect on systemic inflammation [23,24].

**Figure 5:**
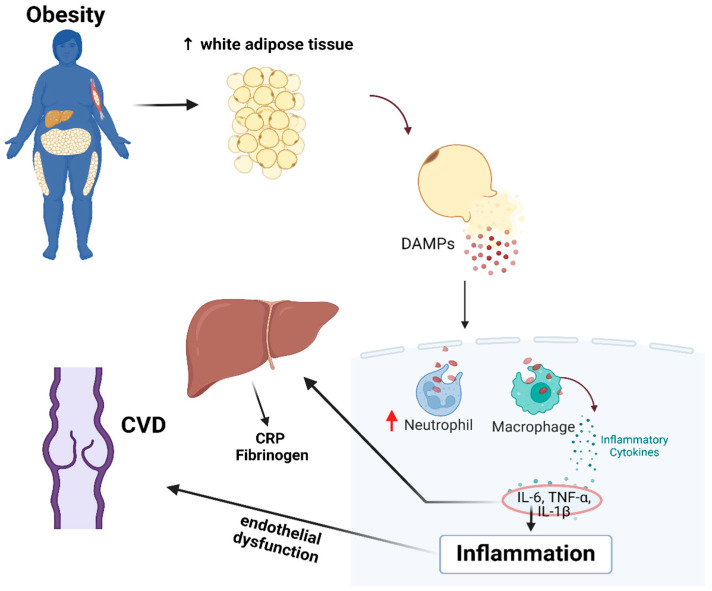
Interplay between Obesity, Inflammation and CVD. Abbreviations: DAMPs (Damage-Associated Molecular Patterns); CRP (C-reactive Protein); CVD (Chronic Venous Disease); TNF-α (Tumor Necrosis Factor-alpha); IL-6 (Interleukin-6); IL-1β (Interleukin-1 beta). Created in BioRender. Petrascu, F. (2024) https://BioRender.com/g28w649 (accessed on 8 September 2024).

Figure 5 emphasizes the interplay between obesity, inflammation and CVD. Obesity is an important risk factor for all types of lower limb venous disease, and obese patients with lower limb venous disease are more likely to be symptomatic [24]. Due to the association of high CRP levels with the development of atherosclerotic lesions [25] and because CRP mediates tissue fibrosis in several cardiovascular diseases [26], it may also be suspected that this characteristic may have an impact on the vasa vasorum from the venous adventitia, accelerating the processes of phlebosclerosis [16,27]. Considering the inflammatory character of CVD [28], as well as the pro-inflammatory status induced by obesity [29,30], it can be considered that the association between CVD and obesity leads to a faster progression of venous insufficiency. This is primarily due to the shared underlying factor, inflammation, which contributes to an increased disease severity and a heightened risk of complications.

A possible mechanism of how obesity could exacerbate CVD is shown in Figure 5. The adipose tissue surplus may be associated with an elevated release of damage-associated molecular patterns (DAMPs) from stressed or damaged cells within the adipose tissue [23,31]. This, in turn, could be correlated with a higher influx of neutrophils and the polarization of macrophages to the pro-inflammatory type (M1). An increase in neutrophils and M1 macrophages could be associated with the continuous production of pro-inflammatory cytokines, contributing to the inflammatory state, which, over time, becomes systemic [24]. These immune cells may contribute to inflammation, which could be linked to venous wall damage and endothelial dysfunction [31,32]. These changes, correlated with microcirculatory impairment, may contribute to the progression of CVD by affecting the vasa vasorum from the venous adventitia and subsequently leading to venous wall fibrosis [17,31].

Pro-inflammatory cytokines, particularly interleukin-6 (IL-6), play a crucial role in regulating the hepatic production of acute phase proteins, such as fibrinogen and CRP, which can be valuable parameters to determine the state of the inflammation [32] (see Figure 4).

The relationship between a higher BMI (obesity) and the progression of CVD underscores the importance of managing obesity as part of the CVD treatment protocol. Inflammation serves as a critical link between obesity and CVD, influencing the disease course and severity. Considering that the excess adipose tissue may contribute to chronic systemic inflammation, which could be associated with poorly healing surgical wounds [33,34,35], postponing surgical intervention may be advisable in obese patients. According to literature data, the outcomes progressively worsened with a BMI > 35 kg/m^2^ for patients undergoing CVD treatment, and the treatment outcomes for patients with a BMI ≥46 kg/m^2^ were so poor that weight loss management should be considered before offering CVD treatment [36].

Also, patients with obesity experienced prolonged pain and more infections after endo-venous thermal ablation (ETA) [37]. Despite the issues and concerns raised by the obese status of the patient, venous reflux ablation remains a goal in preventing further complications like venous leg ulcers or great saphenous vein aneurysm development [38,39]. Some sources claim that ETA for varicose vein treatment remains effective and safe, independent of the patient’s BMI [37]. In addition to diet [40] and exercises in order to lose weight, venous active drugs (VADs) medication and compression therapy should be recommended [41,42]. Minimally invasive procedures like foam sclerotherapy bring a significant success rate after the first session and third-month follow-up with high patient satisfaction [43]. Also, we should consider the fact that the latest data revealed that the improvement of the microcirculatory parameters in patients suffering from CVD is crucial tolong-term good outcomes [44]. Considering the delayed response to treatment of obese patients, largely due to the pronounced pro-inflammatory status, VADs active on the microcirculation should be associated with interventional/surgical treatment and compression therapy [45,46] and used long-term after surgery in all the cases [47]. The available evidence also supports that compression therapy is effective in preventing/reducing further CVD complications and can be long-term used in patients who have surgery contraindications [48]. Therefore, a thorough understanding and assessment of the inflammatory status in these patients not only helps in staging the disease more accurately but also helps in predicting possible complications and tailoring interventions accordingly.

## 5. Conclusions

Our findings demonstrate that age, CRP, fibrinogen, and ANC are significant predictors of CVD progression. While BMI showed a positive correlation with CVD severity, it did not emerge as an independent predictor once inflammatory markers were considered. However, the consistent increase of CRP and fibrinogen with BMI suggests that obesity contributes indirectly to CVD progression by promoting systemic inflammation. This highlights the importance of addressing inflammation as part of the therapeutic strategy in managing obese-CVD patients.

Additionally, our predictive model, which incorporated age, BMI, CRP, fibrinogen, and ANC, demonstrated high accuracy in predicting severe CVD. This model emphasizes the importance of including both clinical and inflammatory parameters in predicting disease outcomes. The model performance highlights the importance of using a multifactorial approach to tailor treatment strategies and improve patient outcomes in CVD.

Incorporating anti-inflammatory interventions alongside conventional treatments may offer a more comprehensive and personalized approach to improve the outcome of patients with severe CVD. Emerging therapies, such as anti-IL-6 treatments that have shown effectiveness in reducing inflammation in conditions like rheumatoid arthritis, hold promise for CVD management but require further clinical validation [48].

## Figures and Tables

**Table 1:** Clinical and paraclinical characteristics of the study group divided by CVD severity.

Variables	Category	Mean (SD) or N (%)				
		Total N = 619 (100%)	Mild N = 251 (40.55%)	Moderate N = 214 (34.57%)	Severe N = 154 (24.88%)	*p*-Value
Age		57.92 (10.39)	54.51 (8.66)	56.30 (9.64)	65.73 (10.01)	<0.001 ^a,^***
Sex	Female	423 (68.34%)	186 (74.10%)	128 (59.81%)	109 (70.78%)	0.003 ^b,^**
	Male	196 (31.66%)	65 (25.90%)	86 (40.19%)	45 (29.22%)	
BMI		28.60 (4.76)	26.75 (3.44)	30.04 (5.26)	29.65(4.92)	<0.001 ^a,^***
Obesity	I	142 (22.94%)	40 (15.93%)	68 (31.77%)	34 (22.07%)	<0.001 ^b,^***
	II	72 (11.63%)	1 (0.39%)	45 (21.02%)	26 (16.88%)	<0.001 ^b,^***
	III	5 (0.08%)	0 (0%)	1 (0.46%)	4 (2.59%)	<0.001 ^b,^***
RBC		4.603 (0.475)	4.622 (0.423)	4.682 (0.428)	4.464 (0.578)	<0.001 ^a,^***
WBC		7.307 (2.198)	6.926 (1.886)	7.465 (2.309)	7.710 (2.416)	<0.001 ^a,^***
ANC		4.539 (1.883)	3.979 (1.484)	4.693 (1.787)	5.240 (2.287)	<0.001 ^a,^***
ALC		1.965 (0.732)	2.084 (0.755)	1.979 (0.668)	1.750 (0.738)	<0.001 ^a,^***
AEC		0.220 (0.213)	0.196 (0.168)	0.229 (0.215)	0.246 (0.265)	0.127 ^a^
ABC		0.045 (0.026)	0.041 (0.023)	0.048 (0.026)	0.046 (0.029)	0.031 ^a,^*
AMC		0.432 (0.214)	0.411 (0.192)	0.440 (0.205)	0.456 (0.255)	0.212 ^a^
PLT		249.060 (77.334)	251.125 (73.047)	236.397 (71.062)	263.292 (89.292)	0.009 ^a,^**
ESR		16.651 (12.100)	9.701 (4.681)	18.074 (11.495)	26 (14.198)	<0.001 ^a,^***
CRP		10.442 (11.397)	5.098 (4.540)	9.052 (6.749)	21.083 (16.251)	<0.001 ^a,^***
Fibrinogen		363.142 (105.666)	318.980 (78.868)	374.383 (110.757)	419.500 (106.230)	<0.001 ^a,^***
PT		12.398 (7.999)	12.518 (10.614)	12.240 (6.133)	12.420 (4.684)	0.64 ^b^
INR		1.070 (0.379)	1.029 (0.177)	1.079 (0.498)	1.123 (0.422)	0.024 ^b^
aPTT		31.442 (5.434)	31.668 (5.241)	31.443 (4.757)	31.072 (6.530)	0.210 ^a^
CK		117.735 (64.964)	127.802 (63.474)	118.934 (64.956)	99.662 (63.974)	<0.001 ^a,^***
CK-MB		18.473 (13.298)	17.908 (10.511)	18.341 (15.181)	19.577 (14.529)	0.971 ^a^
Fasting glucose		103.513 (16.057)	102.035 (16.560)	100.766 (12.918)	109.740 (17.545)	<0.001 ^a,^***
ALT		29.562 (16.191)	31.195 (19.058)	31.677 (15.515)	23.961 (9.380)	<0.001 ^a,^***
AST		23.078 (13.464)	23.818 (18.807)	23.574 (8.483)	21.181 (7.016)	0.054 ^a^

Abbreviations: ^a^ Kruskal–Wallis H test *p*-value; ^b^ Chi-squared test *p*-value; “***”: *p*-value ≤ 0.001; “**”: 0.001 < *p*-value ≤ 0.01; “*”: 0.01 < *p*-value ≤ 0.05; “ns”: 0.1 < *p*-value ≤ 1 (not significant). BMI—body mass index, RBC—red blood cell count, WBC—white blood cell count, ANC—absolute neutrophil count, ALC—absolute lymphocyte count, AEC—absolute eosinophil count, ABC—absolute basophil count, AMC—absolute monocyte count, PLT—platelet count, ESR—erythrocyte sedimentation rate, CRP—C-reactive protein, PT—prothrombin time, aPTT—activated partial thromboplastin time, CK—creatine kinase, CK-MB—creatine kinase-MB, ALT—alanine aminotransferase, AST—aspartate aminotransferase.

**Table 2:** Correlation between clinical predictive factors and severe form.

Risk Factor	Severe Form			
	Spearman Correlation		Univariate Logistic Regression	
	Rho	*p*-Value	cOR(95%CI)	*p*-Value
Age	0.411	<0.001 ***	1.116(1.092–1.142)	<0.001 ***
Female sex	0.030	0.453	1.165(0.787–1.745)	0.453
BMI	0.109	0.006 **	1.062(1.023–1.104)	0.002 **

Abbreviations: Rho—Pearson correlation coefficient, cOR—crude odds ratio for logistic univariate regression; 95% CI, 95% confidence interval; “***”: *p*-value ≤ 0.001; “**”: 0.001 < *p*-value ≤ 0.01; “*”: 0.01 < *p*-value ≤ 0.05; “ns”: 0.1 < *p*-value ≤ 1 (not significant).

**Table 3:** Correlation between paraclinical predictive factors and severe form.

Predictive Factor	Severe Form			
	Spearman Correlation		Univariate Logistic Regression	
	Rho	*p*-Value	cOR(95%CI)	*p*-Value
RBC	−0.148	<0.001 ***	0.435(0.292–0.643)	<0.001 ***
WBC	0.078	<0.001 ***	1.113(1.026–1.207)	0.009 **
ANC	0.189	<0.001 ***	1.288(1.169–1.424)	<0.001 ***
ALC	−0.200	<0.001 ***	0.537(0.395–0.715)	<0.001 ***
AEC	0.027	0.497	2.003(0.889–4.400)	0.086
AMC	0.031	0.430	1.926(0.842–4.347)	<0.001 ***
PLT	0.093	<0.001 ***	1.003(1.001–1.005)	0.009 **
ESR	0.473	<0.001 ***	1.089(1.070–1.110)	<0.001 ***
CRP	0.456	<0.001 ***	1.140(1.112–1.171)	<0.001 ***
Fibrinogen	0.324	<0.001 ***	1.007(1.005–1.009)	<0.001 ***
PT	0.182	<0.001 ***	1.001(0.973–1.022)	<0.001 ***
INR	0.184	<0.001 ***	1.506(0.979–2.422)	0.064
aPTT	−0.066	0.097 **	0.983(0.948–1.017)	0.3298
CK	−0.207	<0.001 ***	0.993(0.989–0.996)	<0.001 ***
CK-MB	−0.009	0.811	1.008(0.994–1.020)	0.242
Fasting glucose	0.186	<0.001 ***	1.032(1.020–1.044)	<0.001 ***
ALT	−0.199	<0.001 ***	0.955(0.937–0.972)	<0.001 ***
AST	−0.069	<0.001 ***	0.979(0.957–0.997)	0.042

Abbreviations: Rho—Pearson correlation coefficient, cOR—crude odds ratio for logistic univariate regression; 95% CI, 95% confidence interval; “***”: *p*-value ≤ 0.001; “**”: 0.001 < *p*-value ≤ 0.01; “*”: 0.01 < *p*-value ≤ 0.05; “ns”: 0.1 < *p*-value ≤ 1 (not significant).

**Table 4:** Multivariate correlation between predictive factors and severe form.

Variables	aOR(95% CI)	*p*-Value
Age	1.101(1.072–1.130)	<0.001 ***
BMI	0.764(0.702–0.830)	<0.001 ***
CRP	1.218(1.164–1.275)	<0.001 ***
Fibrinogen	1.003(1.0002–1.0058)	0.033 *
ANC	1.333(1.170–1.520)	<0.001 ***

Abbreviations: aOR—adjusted odds ratio for logistic multivariate regression; 95% CI, 95 percent confidence interval; “***”: *p*-value ≤ 0.001; “**”: 0.001 < *p*-value ≤ 0.01; “*”: 0.01 < *p*-value ≤ 0.05; “ns”: 0.1 < *p*-value ≤ 1 (not significant).

**Table 5:** Multivariate correlation between predictive factors and combined moderate or severe CVD forms.

Variables	aOR(95% CI)	*p*-Value
Age	1.040(1.019–1.061)	<0.001 ***
BMI	1.024(0.973–1.078)	0.357
CRP	1.133(1.083–1.184)	<0.001 ***
Fibrinogen	1.003(1.0011–1.0057)	0.003 **
ANC	1.246(1.110–1.399)	<0.001 ***

Abbreviations: aOR—adjusted odds ratio for logistic multivariate regression; 95% CI, 95 percent confidence interval; “***”: *p*-value ≤ 0.001; “**”: 0.001 < *p*-value ≤ 0.01; “*”: 0.01 < *p*-value ≤ 0.05; “ns”: 0.1 < *p*-value ≤ 1 (not significant).

**Table 6:** Distribution of BMI Categories among CVD Severity Groups.

BMI Distribution	Mild CVD (N = 251)	Moderate CVD (N = 214)	Severe CVD (N = 154)
Normal weight	70 (27.89%)	49 (22.89%)	27 (17.53%)
Overweight + Obese	181 (72.11%)	165 (77.1%)	127 (82.46%)

## Data Availability

The datasets used and/or analyzed during the current study are available from the corresponding author due to ongoing further determinations.
